# Determinants of resistance to VEGF-TKI and immune checkpoint inhibitors in metastatic renal cell carcinoma

**DOI:** 10.1186/s13046-021-01961-3

**Published:** 2021-06-07

**Authors:** Revati Sharma, Elif Kadife, Mark Myers, George Kannourakis, Prashanth Prithviraj, Nuzhat Ahmed

**Affiliations:** 1 Fiona Elsey Cancer Research Institute, Ballarat, Victoria 3350 Australia; 2 Federation University Australia, Ballarat, Victoria 3350 Australia; 3 The Hudson Institute of Medical Research, Clayton, Victoria 3168 Australia; 4 Department of Obstetrics and Gynaecology, University of Melbourne, Melbourne, Victoria 3052 Australia

**Keywords:** Clear cell renal carcinoma, Metastatic renal cell carcinoma, Vascular endothelial growth factor, tyrosine kinase inhibitor, Sunitinib, Hypoxia, Epithelial mesenchymal transition

## Abstract

Vascular endothelial growth factor tyrosine kinase inhibitors (VEGF-TKIs) have been the mainstay of treatment for patients with advanced renal cell carcinoma (RCC). Despite its early promising results in decreasing or delaying the progression of RCC in patients, VEGF-TKIs have provided modest benefits in terms of disease-free progression, as 70% of the patients who initially respond to the treatment later develop drug resistance, with 30% of the patients innately resistant to VEGF-TKIs. In the past decade, several molecular and genetic mechanisms of VEGF-TKI resistance have been reported. One of the mechanisms of VEGF-TKIs is inhibition of the classical angiogenesis pathway. However, recent studies have shown the restoration of an alternative angiogenesis pathway in modulating resistance. Further, in the last 5 years, immune checkpoint inhibitors (ICIs) have revolutionized RCC treatment. Although some patients exhibit potent responses, a non-negligible number of patients are innately resistant or develop resistance within a few months to ICI therapy. Hence, an understanding of the mechanisms of VEGF-TKI and ICI resistance will help in formulating useful knowledge about developing effective treatment strategies for patients with advanced RCC. In this article, we review recent findings on the emerging understanding of RCC pathology, VEGF-TKI and ICI resistance mechanisms, and potential avenues to overcome these resistance mechanisms through rationally designed combination therapies.

## Background

Kidney cancer is the ninth most common cancer in men and fourteenth in women. In 2018, there were 400,000 new cases around the globe [1]. Renal cell carcinoma (RCC) makes up to 95% of renal malignancies [2]. RCC arises from the renal tubular epithelium, which lines the proximal convoluted tubules and constitutes of very small tubes in the kidney responsible for transporting urine. According to the 2012 consensus conference of the International Society of Urological Pathology (ISUP), there are 15 subtypes of RCC with diverse genetic and epigenetic characteristics, of which clear cell RCC (ccRCC) occurs most frequently (80%). Papillary RCC (10–15%) and chromophobe RCC (5%) are the common remaining histologic subtypes [3]. Around 50% of RCC is detected incidentally with one-quarter of the patients diagnosed with metastatic disease and another 30% that relapse and develop metastatic RCC (mRCC) after undergoing curative nephrectomy. These patient groups are considered at high risk of death due to RCC [4]. The morbidity and mortality rates of advanced RCC are high, with a five-year survival rate of only 18% [5]. All subtypes of RCC are innately resistant to traditional cancer treatments, such as chemotherapy and radiotherapy.

## RCC histology

**Fig. 1:**
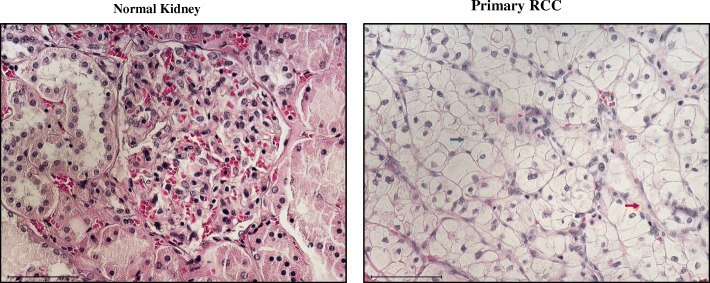
Haematoxylin and eosin (H and E) stained paraffin embedded ccRCC section and its corresponding normal kidney tissue. Histopathological slides showing H and E images of normal kidney with well-defined glomerulus and tubules, and conventional ccRCC with typical histological appearance of epithelial nests of large uniform cells with clear cytoplasm and distinct cell membrane (blue arrow). Delicate branches of blood vessels (red arrow) surround the nests of cells. Magnification: 40X, Scale bar: 75 μm

The gross morphological appearance of RCC varies between tumour types. In general, most RCC presents with areas of extensive network of blood vessels with cysts containing watery fluid and areas of cancer cells (Fig. 1). The ccRCC stores glycogen and lipids and the cells contain clear cytoplasm and a central nucleus encompassed by an intact plasma membrane. Due to the extensive vascular network, the stroma in most cancer cells shrinks, the surrounding parenchyma is constricted, and the tumour is confined to capsular structures [6]. Non-clear cell RCC is a group of diseases, each with different histologic subtypes and different clinical course and outcomes. The most common are papillary RCC (pRCC) and chromophobe RCC (chRCC). pRCC, which show a papillary pattern, although tubular structures or solid growth patterns can be seen but are rare. In the case of chRCC, the growth pattern is often solid, comprised of sheets of tumour cells containing long linear parallel vessels in contrast to the thin delicate network of vessels of ccRCC [7].

## Genetic alterations in RCC

**Fig. 2:**
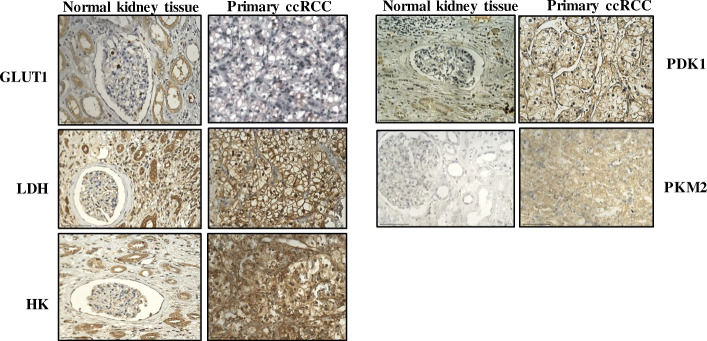
Representative immunohistochemistry images of RCC patient tumour sections and adjacent normal kidney tissues stained with HIF-1α target genes GLUT1, HK, LDH, PDK1 and PKM2. Tumours/tissues were stained as described previously [14]. GLUT1 (abcam: ab652): negative staining in glomerulus and moderate cytoplasmic staining in the tubules of adjacent normal kidney tissue while RCC tissues shows a distinct membranous staining. HK (OriGene Technologies, Inc.; AM05641PU-S): adjacent normal kidney tissue shows strong cytoplasmic staining in the tubules and low cytoplasmic staining in the glomerulus while RCC demonstrates a strong cytoplasmic and membranous staining. LDH (abcam; ab52488): adjacent normal kidney is strongly positive for cytoplasmic staining in the tubules and low cytoplasmic staining in the glomerulus; RCC tissues presents an intense positive membranous and nuclear staining. PDK1 (GeneTex; GTX60386): adjacent normal kidney is negative in glomerulus staining and shows very low cytoplasmic staining in the tubules while RCC tissues illustrates distinct membranous and moderate cytoplasmic staining. PKM2 (abcam; ab150377) staining is negative in the adjacent normal kidney tissue while uniform moderate cytoplasmic staining is present in the RCC tissue. Magnification: 40X, Scale bar: 75 μm. Representative images of *n* = 5 tissues for each antibody

Genetic alterations are common in RCC and usually involve loss of tumour suppressor genes by deletion or functional inactivation or by hypermethylation of the gene promoter [8]. Classically, 70% of ccRCC carry a mutation in the *von Hippel-Lindau* (*VHL*) gene, which encodes VHL protein (pVHL) [9, 10]. pVHL exerts its tumour suppressing function by binding to and mediating the degradation of hypoxia-inducible factor (HIF). The development of RCC in most cases occurs by deletion or mutation on both alleles of *VHL* [9]. The loss or mutation in *VHL* results in the inactivation of pVHL, leading to the activation and accrual of HIF proteins (HIF-1α, HIF-α and HIF-3α) and transcription of downstream target genes [4, 10]. The activation of HIF target genes is fundamental to the pathogenesis of RCC due to their role in promoting angiogenesis, tumour cell survival, proliferation, disease progression, glucose metabolism, and metastatic spread. HIF is also composed of a β subunit (ΗΙF-1β). HIF-1β is stable and constitutively expressed, whereas HIF α subunits are highly unstable and are controlled by cellular oxygen levels. Under normoxic conditions, prolyl 2-oxoglutarate-dependent Fe^2+^ dioxygenases PHD1, PHD2, and PHD3 hydroxylate the two conserved proline residues in HIFα subunits. The hydroxylated proline residues are targets of VHL/E3 ubiquitin ligase complex, resulting in the protease-mediated degradation of HIFα. However, during hypoxia, PHDs cannot hydroxylate HIFα, leading to their stabilisation and activation of downstream target genes, which mostly regulate the expression of angiogenic and tissue remodelling proteins [11]. Stabilised HIF-1α also enhances the expression of glycolytic enzymes lactate dehydrogenase (LDH), pyruvate dehydrogenase kinase (PDK), and other glycolysis-related genes, such as glucose transporter-1 (GLUT-1) and hexokinase (HK), which consequently increase glucose uptake and glycolysis, thus reducing the carbon flux through the tricarboxylic acid (TCA) cycle and oxidative phosphorylation in RCC [12, 13]. Enhanced expression of HIF-1α target genes GLUT1, HK, LDH, PDK1 and PKM2 in ccRCC compared to matching adjacent normal kidney tissues are represented in Fig. 2.

Hence, HIF-1α is a vital metabolic checkpoint for RCC and is essential for the development of RCC in vivo. Recent studies have shown that inactivation of both HIF-1α and HIF-2α hampers the development of ccRCC in mouse models, suggesting that both HIFα genes may be crucial for ccRCC initiation and progression [15]. However, 30–40% of clinically diagnosed ccRCC lack the expression of HIF-1α, suggesting that HIF-1α may act as a tumour suppressor gene in those scenarios where its expression may be mandatory for initial development but lost as the tumour progresses [16]. By contrast, HIF-2α plays a critical role in RCC progression through its activating effects on c-Myc, epidermal growth factor receptor (EGFR), cyclin D, tumour protein p53, and mammalian target of rapamycin (mTOR) oncogenes, resulting in enhanced cell cycle progression and tumour growth [16–18]. Hence, ccRCC cases are divided into two groups. The first group has both HIF-1α and HIF-2α expressed to drive tumour progression, whereas in the second group, the effect of only HIF-2α prevails, particularly in vivo in rapidly proliferating tumours where access to nutrients is limited for tumour cells, resulting in enhanced tumour cell proliferation/angiogenesis and poor patient prognosis [16].

Next-generation sequencing has identified genes other than *VHL* that are commonly altered in RCC. Polybromo 1 (*PBRM1) (41%), BRCA1-*associated protein 1 *(BAP1) (15%), and* SET domain containing 2 histone lysine methyltransferases *(SETD2) (19%)* have been mapped to chromosome 3p, similar to *VHL* [19]*. PBRM1* is a subunit of SWI/SNF chromatin remodelling complex, BAP1 encodes the histone deubiquitinating enzyme BRCA1-associated protein, and SETD2 is a histone methyltransferase [20]. Studies have shown that other tumour suppressor genes, such as Wilms tumour 1 gene (*WT1*), the phosphatase and tensin homolog (*PTEN*), and tumour protein *p53,* are also involved in the pathology of RCC [21, 22]. Among these genes, *p53* mutation has been shown as a prognostic indicator for RCC, with increased frequency of *p53* mutation reported with increasing grades and stages of RCC [23]. In this context, the correlation of *p53* mutation with disease-specific survival was reported in RCC patients [24].

## Epithelial-mesenchymal transition in ccRCC

**Fig. 3:**
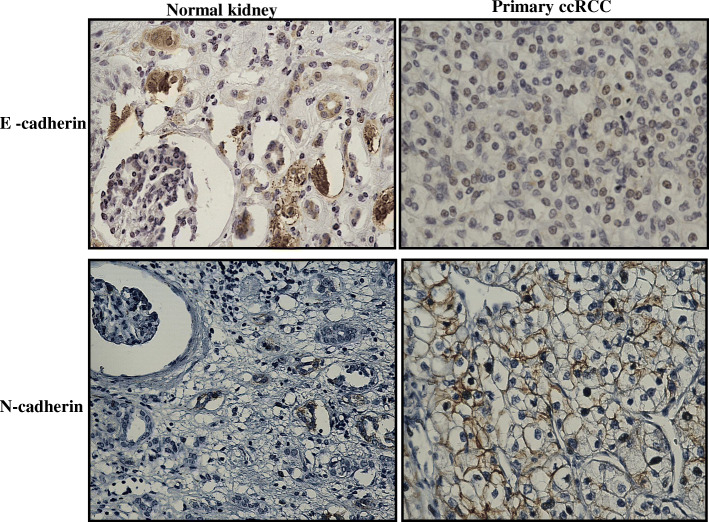
Representative immunohistochemistry images of RCC patient tumour sections and adjacent normal kidney tissues stained with E-cadherin and N-cadherin. Immunohistochemistry was performed as described in Fig. 2. E-cadherin (Cell Signaling; 14,472), images show adjacent normal kidney tissue to be slightly positively stained in the tubules while RCC tissue is negative for any staining. N cadherin (Cell Signaling; 13,116): increase in the membranous expression of N-cadherin in the RCC tumour compared to normal adjacent kidney tissue. Magnification: 40X, Scale bar: 75 μm. Representative images of *n* = 5 tissues for each antibody

Epithelial-mesenchymal transition (EMT) is an embryonic development process that cancer cells utilize, whereby epithelial cells lose their epithelial polarity and attain a mesenchymal phenotype and shape in order to detach from primary sites to gain entry into surrounding tissue vasculatures for re-localization and spread into surrounding or distant sites [25]. The process is triggered by various stimuli received by the cancer cells from the tumour microenvironment, one of which is hypoxia-mediated HIF1-α activation, which plays a critical role in the initiation and orchestration of EMT [25]. The dissemination of cancer cells is facilitated by the loss of epithelial cell adhesion molecule E-cadherin and upregulation of E-cadherin repressors such as Slug, Snail, ZEB, and Twist, which are the hallmarks of the EMT process. An immunohistochemistry study on ZEB2 expression in 116 RCC patients demonstrated high ZEB2 expression in RCC tumours that correlated with poor overall survival (OS) and progression-free survival (PFS) in RCC patients [26]. Similarly, enhanced Snail expression was frequent in high-grade RCC and associated with poor OS and PFS in RCC patients [27]. A recent study correlated EMT with an increased risk of recurrence and poor OS in RCC patients based on the expression of DCLK1 (a serine/threonine kinase involved in microtubule-mediated neuronal migration and morphogenesis) in RCC tumours [28]. In addition, DCLK1 was shown to be overexpressed and deregulated in > 93% of RCC tumours, and its knockdown by siRNA in RCC cells resulted in decreased expression of EMT and cancer stem cell (CSC) markers [29]. Further, the scoring of EMT based on the identification of spindle-shaped cells in tumours obtained from 47 RCC patients after nephrectomy was correlated with a shorter OS of 3–6 months in 96.4% patients compared to a longer OS of > 6 months in 42.1% patients in whom spindle-shaped cells were absent [30]. A multivariate analysis of the expression levels of Clusterin, Twist, and C-reactive protein (CRP) in the tumours of 116 RCC patients obtained at nephrectomy independently predicted disease recurrence and recurrence-free survival established by the positive expression of each independent factor present in individual patient. Disease recurrence was observed in 7.7% of patients who were negative for any risk factor, 31.5% in patients who had one or two risk factors, and 60.9% of patients with three or four risk factors [31]. RCC tumour stage and histological grade, as well as sarcomatoid differentiation, are influenced by the expression of the transcription factor Snail. The conversion of ccRCC into sarcomatoid tumour is regulated by EMT by triggering N-cadherin expression, dissociation of β-catenin from the cell membrane, and increased expression of Snail and Sparc proteins [32, 33]. A recent study used an integration of omics and cellular/molecular biology assays on 26 RCC patient samples to demonstrate a link between fibrosis and EMT correlating that to worse patient survival [34]. The above studies clearly indicate that EMT is a crucial driving force in RCC progression. Besides hypoxia-mediated HIF-1α activation, several cytokines also contribute to the orchestration of EMT during RCC progression. Among these, IL-6, IL-8, IL-15, and tumour necrosis factor α (TNF-α) play a prominent role in EMT facilitation via Akt/GSK-3β/β-catenin signalling pathway [35–38]. Chronic oxidative stress also induces EMT characteristics in RCC cells [39]. Downregulation of the microRNA (miRNA)-200 family, which includes miR-200a/b/c, miR-141 and miR-429, is also involved in the EMT process in RCC [40]. A recent paper has shown that an immune suppressor cyclosporine in combination with transforming growth factor β (TGFβ) is able to induce EMT and CSC-like phenotypes in RCC cells [41]. Figure 3 demonstrates indication of EMT in RCC by illustrating enhanced staining of EMT-related N-cadherin and low expression of E-cadherin in ccRCC tumours compared to adjacent normal kidney tissues.

## Cancer stem cells (CSCs) in RCC

CSCs constitute a minor population of cells within tumours with a remarkable ability for tumour initiation and sustenance through infinite capacity for self-renewal and multi-lineage differentiation towards heterogeneous progenies [42]. RCC is known to be a heterogeneous tumour with the existence of both intra- and inter-heterogeneity. Heterogenous cell populations are functionally and phenotypically distinct and therefore display varying degrees of response and sensitivity to drugs, reducing the likelihood of treatment success. As such, CSC-like cells may be important determinants of clinical resistance and patient outcomes [43, 44]. Further to that, the heterogeneity in CSCs may be modulated by a diverse range of factors, including genetic mutations, epigenetic changes, stimulus from the tumour microenvironment due to cell-cell interaction, exposure to different cytokine milieus, and hypoxia [45, 46].

**Fig. 4:**
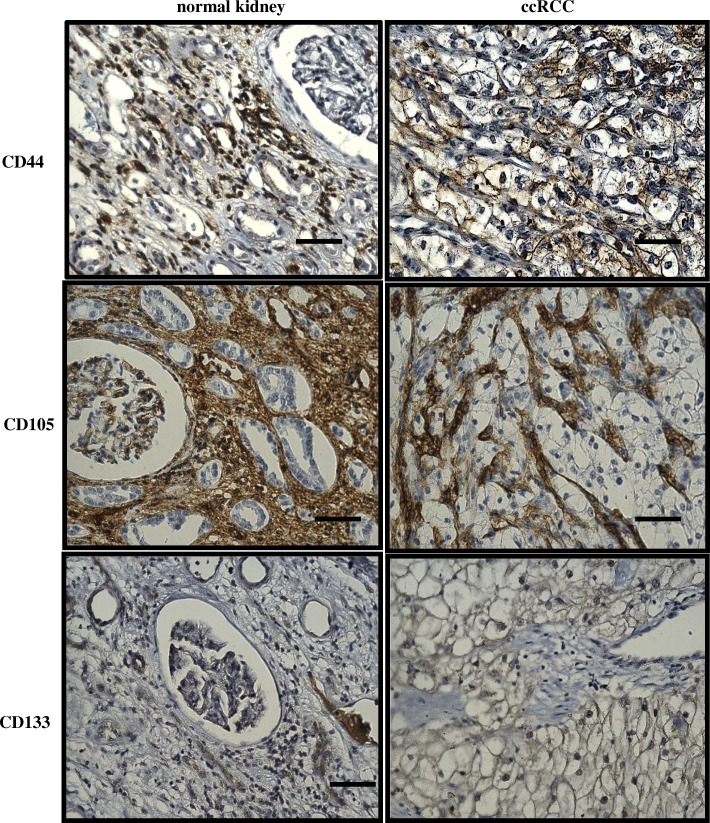
Representative immunohistochemistry images of RCC patient tumour sections and adjacent normal kidney tissues stained with CSC markers CD44, CD105 and CD133. Immunohistochemistry was performed as described in Fig. 2. CD44 (abcam; ab51037): normal kidney tissue shows negative staining in glomerulus and moderate cytoplasmic staining in the tubules; while RCC tissues shows distinct membranous and moderate cytoplasmic staining. CD105 (abcam; ab169545): adjacent normal kidney tissue shows strong cytoplasmic staining in the tubules and low cytoplasmic staining in the glomerulus; RCC tissues shows an intense positive membranous staining. CD133 (abcam; ab19898): adjacent normal kidney showing negative staining; RCC tumour shows a specific membranous staining. Magnification: 40X, Scale bar: 75 μm. Representative images of *n* = 5 tissues for each antibody

The mere observation that cancers can arise long after initial exposure to carcinogens implies that the carcinogenic event imposed by oxidative, genotoxic, or cytotoxic stress leading to damage-associated molecular pattern (DAMP) response may persist in the residual long-lived slowly proliferating stem cell population for an indefinite period ranging from months to years. This dormant response eventually triggered by unknown mechanism(s) gives rise to generations of daughter and differentiated cells, resulting in recurrent tumour masses [47]. Hence, recurrent or relapsed tumours that arise from CSCs consist of CSCs and a mixed population of cells, which create the full heterogeneous phenotype of the tumour. The induction of EMT giving rise to CSC-like cells was first shown in breast cancer, in which stimulation by TGFβ resulted in both EMT and CSC-like cells [48]. Consistent with this result, the introduction of mesenchymal markers Twist or Snail, responsible for the suppression of the epithelial adhesion molecule E-cadherin, led to an increase in the number of CSC-like cells in breast cancer [49]. Hypomethylation of genes specific for the transcription stem cell programme leads to EMT in cancer cells [50]. Moreover, E-cadherin transcriptional repressors Snail and Slug enforce CSC-like phenotypes and chemoresistance in ovarian cancer cells [51]. Recent studies have shown the existence of a side population (SP) cells in RCC tumours, a distinct type of CSCs, detected by the use of Hoechst 33342 dye (DNA binding dye) that displays a unique pattern by fluorescence-activated cell sorting (FACS) [52]. However, no general applicable panel of markers for CSCs has been identified in RCC, and the characterization of putative CSCs varies in individual studies and is mostly based on their functional parameters. RCCs have been shown to display diverse CSC markers (such as CD44, CD133, CD105, CXCR-4, Oct4, Nanog, Klf4, and LIN28) and have a high expression of the ATP-binding cassette family of transporter proteins, such as MDR1 (P-glycoprotein) and ABCB transporters [53]. However, the proportion of cancer cells expressing different CSC markers remains uncertain and may not always reflect a true proportion of CSCs or have the CSC-like phenotype as described in other tumours [44, 54]. Furthermore, hypoxia plays a critical role in the conservation of EMT and CSC features in solid tumours. In RCC, hypoxia-induced HIF-1α promoted EMT in RCC cell lines through increased expression of ZEB1 and ZEB2 and E2A immunoglobulin enhancer-binding factors E12/E47 (TCF3), which suppressed E-cadherin expression, leading to the attainment of a mesenchymal phenotype in these cells [55]. Tumour-infiltrating macrophages have been shown to induce EMT and CSC-like phenotypes in RCC cell lines and mouse xenografts [56]. Ectopic expression of retinoblastoma binding protein-2 (RBP2) promoted CSC phenotypes through EMT in RCC cells [57]. These observations indicate that the collaboration of EMT and CSC is crucial for RCC progression. Figure 4 demonstrates presence of CD44, CD105 and CD133 positive CSC staining in RCC tumours and adjacent normal kidney tissues.

## Hypoxia and its effect on the tumour microenvironment (TME)

For cancer to adapt to low oxygen, tumour cells aberrantly develop new but defective and leaky blood vessels. The abnormal vasculature, together with the hypoxic microenvironment, promotes angiogenesis and inflammation, all of which lead to tumour progression and treatment resistance. Studies have highlighted that therapy resistance and cancer progression are not only regulated by tumour cells but also by the cells and components encompassing the tumour microenvironment (TME) [58]. Hypoxia induces genetic and proteome changes in tumours and associated cells in TME leading to accelerated cancer progression and induction of a more resistant tumour phenotype [59]. Hypoxia also decreases drug penetration and increase the expression of drug efflux transporters in tumours as well as tumour associated endothelial cells (TECs) [59]. In addition, the hypoxic environment promotes tumour cell glycolysis, which enhances lactic acid production, favouring a low pH TME that suppresses immune cell functions such as proliferation and cytotoxicity [60].

One cell population that thrives under hypoxic conditions in tumours are TECs. These cells are an important component of RCC TME and are key to progression and therapy resistance. Higher levels of circulating endothelial cells have been noted in mRCC patients treated with sunitinib who acquire resistance [61]. Contrary to the popular belief that TECs are homogenous and cannot proliferate in RCC, these cells are capable of hyperproliferation and display metabolic, genetic and morphological abnormalities compared to normal endothelial cells (NECs) [62]. TECs isolated from human tumour xenografts of RCC, melanoma and liposarcoma displayed chromosomal irregularities associated with aneuploidy [63, 64]. These aneuploid TECs in TME were surrounded by pimonidazole-positive areas, indicating association of hypoxia with aneuploidy [64]. The same study showed that aneuploidy could also be induced in NECs in response to hypoxia, which was inhibited by inhibitors of VEGFR2 or reactive oxygen species (N-acetyl-L-cysteine) [64]. These studies indicate that hypoxia modulates phenotypic and genotypic characteristics of TECs via VEGF and ROS expression in TME and may transform NECs to TECs to favour tumour progression.

A recent study has shown TECs isolated from hypoxic highly metastatic tumours contained more aneuploid cells, had enhanced proliferative and invasive capacity and had enhanced mRNA expression of pro-angiogenic (VEGF, VEGFR1/2, HIF-1α) and stemness genes than TECs derived from low metastatic tumours [65]. In addition, TECs from different tumours (melanoma, liposarcoma, RCC, glioma, breast and hepatocellular carcinoma) overexpress proangiogenic growth factors and receptors suggesting an autocrine loop for sustenance in an activated mode in TME [66]. Compared to NECs, TECs are also more resistant to serum starvation and cytotoxic drugs [66–69]. TECs in RCC express PAX2 and HLA-G, two embryonic markers generally expressed in renal tumours [70, 71]. The expression of embryonic markers in RCC TECs may indicate their dedifferentiation status different from adult or tumour stem or progenitor cells [66]. These functional alteration in TECs may result from constitutively activated signalling pathways, such as PI3K/Akt [72], Cox-2 pathways [73] and downregulation of anti-angiogenic factors such as thrombospondin-1 (TSP-1) and endostatin, [72, 74], responsible for the induction of resistance to chemotherapeutic and antiangiogenic drugs.

Further to that, recent studies have shown that CSCs in leukemias, breast and ovarian cancer to differentiate into endothelial cells [75, 76]. In RCC, CD105^+^ and CD133^+^ CSCs were noted to generate endothelial cells in vivo [77, 78]. Considering that hypoxic cancer cells are poorly differentiated and express markers of CSCs [79, 80], it can be postulated that hypoxic RCC with CSC phenotype may have the potential to initiate and promote vasculogenesis to sustain and accelerate tumour growth. In that context, hypoxia induced increased expression of HIF-1α and HIF-2α has been noted in neuroblastoma and glioma CSCs [81, 82]. Both HIF-1α and HIF-2α are also associated with hypoxia-induced expression of CD133 and knocking down of either HIF-1α [83] or HIF-2α [84] was shown to reduce hypoxia-induced CD133 expression in glioma CSCs.

Along with irregular blood vessels, hypoxia can also lead to blocked lymphatic drainage with increased interstitial pressure [59]. This interstitial fluid pressure within the tumour can interfere with tumour cell’s drug uptake by counteracting the passage of drug into the tumour cells. Cancer-associated fibroblasts (CAFs) in the TME are associated with these pressure forces within the tumour, and some studies have successfully demonstrated improved uptake of cytotoxic drugs by targeting CAFs [85]. In mRCC, activation of fibroblast activating protein was shown to induce aggressive phenotype in RCC via CAFs-mediated recruitment of macrophages leading to remodelling of TME [86]. In addition, a recent study has identified a distinct angiogenesis^high^macrophages^low^ fingerprint in a cluster of RCC, which may prove crucial for predicting anti-TKI/anti-angiogenesis treatments [87]. Other studies have also linked CAFs with resistance to antiangiogenic drugs [88, 89]. In addition, TECs have immune regulatory roles as they are directly involved in affecting T cell priming and migration, modulating immune cell trafficking by favouring infiltration in tumours of immune suppressive (such as Tregs, MSDCs, TAMs) rather than immune effector cells (CD8^+^ and CD4^+^ cells) [90]. Hence, it is important to characterise the cancer promoting functions of hypoxia-modulated TECs to develop new strategies targeting TME associated TECs in combination with tumour cells.

RCC has long been recognised as an immunogenic tumour due to a substantial amount of immune cell infiltration in the tumours [91]. However, the mere presence of immune cells in the tumours (TILs) does not indicate that these immune cells are active to mount an anti-tumour response. A recent study on the peripheral blood mononuclear cells (PBMCs) of 90 RCC patients showed increased expression of PD-1 on CD14 bright myelomonocytic cells, effector T cells and natural killer (NK) cells, which correlated with disease stage. The PD-1 expression on immune cells was significantly reduced after surgery of primary tumours [92, 93]. In another study on the PBMC of 40 RCC patients identified CD8^+^PD-1^+^TIM-3^+^Lag3^+^ TILs, CD4^+^ICOS^+^TILs and CD25^+^CD127^+^Foxp3/Helios^+^ GITR^+^Tregs phenotype to be associated with high risk of disease progression after nephrectomy within the same year [94]. In a subsequent study it was shown the patients having high levels of CD8^+^PD-1^+^TIM3^+^LAG3^+^ in PBMCs responded significantly well to nivolumab (anti-PD-1) but not to everolimus in terms of overall and progression-free survival, suggesting a specific therapeutic role of nivolumab in these patients [95]. These data suggests the potential of TIM3 and LAG3 as additional checkpoint inhibitors in RCC management. Very recently, tumour-educated B cells (TEB) within the RCC TME were shown to play a key role in RCC progression and therapy resistance [96, 97].

## The role of non-coding RNAs (miRNAs) in RCC

Recent studies have identified circulating non-coding RNAs such as miRNAs as potential blood-based biomarkers for early-stage diagnosis, prediction of prognosis and treatment response in RCC [98]. Among the different miRNAs described in RCC, miR-210 was shown by several studies to hold promise as a potential early-stage biomarker as its expression level was significantly enhanced in malignant tissues compared to healthy adjacent parenchyma, and its level in the serum of RCC patients was significantly high compared to healthy controls [14, 99–102]. According to these studies, the serum miR-210 levels could differentiated RCC patients from healthy controls however, both sensitivity and specificity of miR-210 varied substantially between the studies. Other studies have shown regulation of miR-210 by hypoxia, showing upregulation of miR-210 in response to hypoxic conditions in RCC cell lines, suggesting a close relationship of miR-210 with RCC development [101–103].

Apart from miR-210, combination of miR-210 and miR-378 provide greater discriminatory ability of identifying RCC patients from healthy individuals [14]. However, significant enhancement in the serum levels of miR-378 levels have been controversial with some studies showing its reduced serum levels in RCC patients compared to healthy controls [104]. Apart from that, combination of miR-378 and miR-451 in serum of RCC patients could provide sensitivity of 81% and specificity of 83% compared to healthy individuals [105].

Further to that, a miR diagnostic signature for RCC patients based on serum expression of different miRNA consisting of miR-378, miR-193a-3p, miR-362, miR-572 and miR-28-5p was developed [104]. However, its clinical utility in patients could not be conclusively analysed as the expression of these miRNAs were not deduced in corresponding tissues. In addition, the expression of several other miRs (extensively discussed in [98]) was noted to be elevated in the serum of RCC patients compared to control individuals but none showed prognostic utility in a clinical setting.

## Treatment of metastatic RCC

Recent advances in understanding the molecular and genetic characteristics of RCC have led to the development of many novel drugs, leading to improved clinical outcomes. Nivolumab, cabozantinib, and lenvatinib plus everolimus have gained Food and Drug Administration (FDA) approval in the last 2 years. In the following sections, we outline the currently understood innate and acquired drug resistance mechanisms in RCC and discuss the current novel approaches used to overcome such resistance.

The selection of treatment for RCC patients depends on the prognostic risk factors. These risk factors guide clinical trial design, patient counselling, and risk-specific treatment decisions. Five prognostic factors, including haemoglobin < lower limit of normal (Normal for men: 13.5–17.5 g/dL and normal for woman: 12–15.5 g/dL), time from diagnosis to systemic treatment < 1 year, calcium > 10 mg/dL, LDH > 1.5x upper limit of normal were correlated with overall survival (OS) in metastatic RCC (mRCC). These factors were integrated into a prognostic risk score called the Memorial Sloan- Kettering Cancer Center (MSKCC) score [106]. However, when VEGF-TKIs revolutionized the treatment options of mRCC, there was a need for a new prognostic score, and the International Metastatic RCC Database (IMDC) was founded. Based on the median OS, the IMDC prognostic score has three risk groups: favourable, intermediate, and unfavourable. Treatment options for mRCC follow the IMDC prognostic risk factors. Table 1 outlines the IMDC criteria for prognostic evaluation.

**Table 1:** IMDC prognostic score risk groups

Number of risk factors	Risk Group	Median overall survival (months) (95% CI)
0	Favorable/Good	43.2 (31.4–50.1)
1 to 2	Intermediate	22.5 (18.7–25.1)
3 to 6	Unfavorable/Poor	7.8 (6.5–9.7)

The above table describes the criteria for the risk groups depending on the number of risk factors and the median OS of the patients. The six risk factors taken into consideration include low Karnofsky performance status (< 80%), low serum hemoglobin, high serum calcium level (> 10.2 mg/dL), increased neutrophil and platelet count (7 × 10^9^/L and 400,000 respectively) and time from diagnosis to the treatment < 1 year

Before the development of advanced therapeutics, such as VEGF-TKIs and immunotherapy, the treatment for mRCC revolved around the use of cytokines such as interleukin 2 and IFN-α, which were effective only in 5–15% of patients [107]. However, cytokine therapy alone was not enough to overcome the complex vascularization and metastatic biology of RCC regulated by HIF-induced downstream angiogenic signalling pathways [108]. Hence, there was a need for antiangiogenic treatment that would target VEGF and mTOR pathways and potentially control angiogenesis to provide better OS and PFS in advanced RCC patients. Many observational studies have validated the significant role of anti-angiogenesis therapy in RCC [109–111]. However, since 2004, the introduction of more target-specific therapies and immunotherapy has created a paradigm shift in the treatment of RCC.

### Angiogenesis inhibitors

Antiangiogenic VEGF-TKIs, such as sunitinib and pazopanib, are currently used as the first-line treatments in RCC. Sunitinib showed a high response rate of 8.3 months of PFS in a multicentre phase II trial [112]. The encouraging objective response based on phase I and phase II trials led to a crucial randomized phase III trial in 750-treatment naïve advanced RCC patients. The results demonstrated superior efficiency of sunitinib (11 months) over IFN-α (5 months) in PFS and supported its use as the first-line treatment for mRCC [113, 114]. The median overall survival in the sunitinib-treated patients was also higher than in the IFN-α group, being 26.4 versus (vs.) 21.8 months, respectively, and the common toxicity demonstrated in patients included hand and foot syndrome, diarrhoea, and hypertension [114]. In phase III trial, pazopanib showed a similar result of a median PFS of 11.1 vs. 2.8 months compared with a placebo in the treatment-naïve subpopulation, and 7.4 vs 4.2 months compared to cytokine pre-treated patients [115]. The overall survival in pazopanib-treated patients was 22.9 months, and the most experienced adverse effects (AEs) were hypertension, vomiting, diarrhoea, anorexia, and hair colour changes [116]. Owing to the similar PFS benefits, two randomized controlled studies COMPARZ trial and PISCES study were undertaken to compare sunitinib and pazopanib to find the optimal first-line therapy. While the primary endpoint of the COMPARZ trial was PFS, the PISCES study assessed patient preference between pazopanib and sunitinib as the primary endpoint. Pazopanib emerged non-inferior to sunitinib in terms of PFS and overall survival, with 70% of patients preferring pazopanib to sunitinib [117, 118]. The two pivotal studies have placed sunitinib and pazopanib at par as standard front-line treatments for mRCC across the world. In terms of direct transferability of these clinical trial results in patient care, many recent retrospective studies have associated sunitinib with better overall survival compared to pazopanib [119–121].

Sorafenib and axitinib are the other VEGF-TKIs that have been tested as first-line treatments in advanced RCC. First introduced in 2004, sorafenib is an antiproliferative and antiangiogenic agent and a multi-target VEGF-TKI against VEGFRs (1–3), platelet derived growth factor-β (PDGRF-β), c-Kit protein (c-Kit), FMS-related receptor tyrosine kinase 3 (FLT-3), Raf kinases (C-Raf, B-Raf), mutant B-Raf, rearranged during transfection (RET), and RET/papillary thyroid carcinomas (PTC) [122, 123]. An open-label phase II trial evaluating sorafenib vs. IFN-α for PFS, overall response, and adverse events was conducted in 189 patients with untreated advanced RCC. It was found that sorafenib did not improve the PFS when compared to IFN-α [124]. However, according to the TARGET trial, a phase III randomized placebo-controlled trial in 903 therapy-failed patients, sorafenib improved progression-free survival (5.5 vs. 2.8 months) [125]. Even though the European Society for Medical Oncology (ESMO) guidelines included sorafenib as a first-line treatment, this was not endorsed by National Comprehensive Cancer Network (NCCN) guidelines [126, 127]. A phase III SWITCH trial showed that there was no significant difference in the PFS between the sequential treatment of sorafenib followed by sunitinib and vice versa. This followed another phase III trial, SWITCH-II, which compared the total progression-free survival (tPFS) between sorafenib-pazopanib (So-Pa) and pazopanib-sorafenib (Pa-So). However, So-Pa did not meet the total PFS (8.6 vs 12.9 months) criterion when compared with Pa-So in 377 randomised patients [128].

Axitinib, a tyrosine kinase inhibitor of VEGFRs 1–3, is used as a second-line option for mRCC. However, it was evaluated as a first-line agent and compared with sorafenib in a phase II trial in 192 patients across 13 countries. The primary endpoint was PFS. Axitinib did not demonstrate an increase in the PFS when compared to sorafenib. Although axitinib did not show any superiority over sorafenib, it is included as a first-line treatment option in NCCN guidelines (category 2A). The guidelines take into consideration that axitinib has demonstrated clinical activity and an acceptable safety profile [126, 129].

Cabozantinib is a small molecule oral VEGF-TKI. The FDA first approved its use in November 2012 to treat metastatic medullary thyroid cancer. It was approved in April 2016 as a second-line drug treatment of patients with RCC who had previously received antiangiogenic therapy [130]. Cabozantinib is different from other VEGF-TKIs as it targets multiple tyrosine kinases implicated in mRCC in addition to VEGFR, such as mesenchymal-epithelial transition factor (MET), anexelekto (AXL), RET, KIT, and FLT3 [131]. Pre-clinical studies have shown an increase in the expression of MET and AXL in RCC tumours when exposed to chronic sunitinib therapy; these are important resistance mechanisms in RCC [57, 132]. This pre-clinical breakthrough gave a strong rationale for cabozantinib to be studied clinically in the METEOR trial. The phase III randomised trial compared cabozantinib and everolimus and included 658 patients who progressed with the cancer after treatment with at least one VEGF-TKI. The primary endpoint was mPFS, whereas the secondary endpoint was OS and overall response rate (ORR) and safety. The study achieved its primary endpoint with cabozantinib showing a superior outcome to everolimus (7.4 vs 3.8 months). The rate of progression of the disease or death was 42% lower in cabozantinib than with everolimus [133]. A follow-up study after 1 year observed an improved median OS of 21.4 months in cabozantinib-treated patients in comparison to 16.5 months with everolimus. The ORR was 17% with cabozantinib vs 3% with everolimus. The most common adverse event noted was hypertension [134].

Similar to the METEOR study, the CABOSUN study was undertaken to compare the clinical benefits of cabozantinib with sunitinib in 157 treatment-naïve patients with intermediate to poor IMDC risk. Patients treated with cabozantinib showed improved PFS (8.6 vs. 5.3 months) and ORR (46% vs. 18%) [135]. A superior OS was achieved in patients treated with cabozantinib; however, it was not significant (26.6 vs. 21.2 months). With cabozantinib, the rate of disease progression or death decreased by 34%. Similar grade 3 or 4 adverse events were observed for patients with cabozantinib and sunitinib and included diarrhoea, fatigue, hypertension, palmar-plantar erythrodysthesia, and hematologic adverse events (67% vs. 68%, respectively) [136]. Based on the CABOSUN results, NCCN and ESMO recommended cabozantinib as a first-line treatment option for patients with poor to intermediate IMDC risk (Category 2A). This recommendation was made at a lower level than the category 1 agents pazopanib, sunitinib, and bevacizumab plus IFN-α [126, 137]. Cabozantinib has recently been shown to have enhanced efficacy in a retrospective cohort study investigating naïve and refractory metastatic non-clear RCC belonging to all IMDC model risk groups [138].

## Immune checkpoint inhibitors

Immunotherapy has been an integral part of RCC treatment for decades. RCC is categorized as an immunogenic tumour based on its response to immunotherapy and high level of T cell infiltration, including dendritic cells, natural killer T cells, macrophages, and memory cells, along with increased cytokine secretion [139]. More than a decade ago, treatment of RCC patients heavily depended on interleukin-2 (IL-2) and IFN-α, which not only yielded a low efficacy and overall response but also was also associated with significant toxicity. Rapid development in immune checkpoint inhibitors in the past decade has helped fill the gaps left by IL-2 and IFN-α.

### Nivolumab and Ipilimumab

Recently, Nivolumab, an anti-programmed cell death protein 1(PD-1) monoclonal antibody, was the first immune checkpoint inhibitor approved by the FDA in 2015 for RCC patients based on a phase III clinical trial CheckMate 025. Nivolumab exploits a negative co-stimulatory signal meant to mitigate T cell receptor (TCR) signalling. The trial compared nivolumab with everolimus and was carried out in 821 patients previously treated with one or two antiangiogenic therapies. The overall survival of the patients treated with nivolumab was significantly higher (25 vs.19.6 months) than those treated with everolimus, and the most common adverse event noted was fatigue. Another less successful checkpoint inhibitor, ipilimumab, designed to reduce the inhibitory effect of cytotoxic T lymphocyte-associated protein 4 (CTLA-4) resulted in significant autoimmune toxicities in 61 patients [140].

Although nivolumab in monotherapy had shown improved overall survival in the CheckMate 025 trial, the median PFS (mPFS) was not much superior to everolimus (4.6 vs. 4.4 months). This observation, along with the fact that ipilimumab had shown limited efficacy and significant toxicity in patients, supported the rationale for combining both nivolumab and ipilimumab. This combination produced objective responses in RCC patients in a pilot study that led to a phase III trial, CheckMate 214, comprising 1096 treatment-naïve patients [141]. The primary endpoints were overall survival, objective response rate, and progression-free survival. After a median follow-up of 25.2 months in intermediate and poor-risk patients, the 18-month OS rate was 75% with the combination immunotherapy and 60% with sunitinib. The median overall survival was not reached with the nivolumab-ipilimumab combination vs. 26 months with sunitinib. The mPFS was 11.6 months in the combined immune checkpoint inhibitors as compared to 8.4 months in sunitinib [142]. Further evaluation of patient-reported outcomes showed fewer symptoms and improved health-related quality of life (HRQoL) with combination therapy than sunitinib in intermediate or poor-risk patients with RCC [143]. The encouraging findings that suggested a superior efficacy of nivolumab and ipilimumab over sunitinib led the FDA to approve this double immune checkpoint blockade in April 2018 for RCC patients with intermediate or poor-risk features.

### mTOR inhibitors

The mammalian target of rapamycin (mTOR), a member of the phosphatidylinositol-3-kinase (PI3K) family, is an important component of intracellular signalling pathways that activates growth factors and regulates cellular metabolism, proliferation, and angiogenesis. Although everolimus and temsirolimus have shown to be effective against RCC in patients, only temsirolimus gained approval to be used as a first-line agent to treat patients with unfavourable risk factors. Temsirolimus was approved after a phase III Global Advanced Renal Carcinoma trial, conducted in 626 treatment naive patients with poor prognostic features. This trial showed greater OS (10.9 months) in comparison with IFN- α alone (7.3 months) or as a combination therapy (8.4 months). The study also reported that temsirolimus had a higher PFS (3.8 months) than IFN- α (1.9 months) [144]. Although these phase III trial results were promising, temsirolimus is not a common treatment method in a regular clinical setting [127]. Interestingly, there are no clear studies comparing temsirolimus with the existing first-line VEGF-TKIs. However, RECORD-3, a phase II study that was conducted in 238 patients, compared the sequence of everolimus followed by sunitinib and vice versa. The overall survival did not support the use of everolimus-sunitinib sequential therapy [145].

### Pembrolizumab plus axitinib

Recently, a phase III trial, was conducted with 861 patients randomly receiving axitinib plus pembrolizumab, a PD-1 inhibitor, and sunitinib monotherapy in previously untreated patients with mRCC [111, 146]. The primary endpoint mPFS was significantly higher with the pembrolizumab plus axitinib combination than with sunitinib (15.1 vs 11.1). The risk of progression or death was reduced by 47% with the combination therapy. This benefit was observed across all IMDC risk groups. The most common grade 3 and 4 AEs in both groups were diarrhoea and hypertension. Based on the benefits and tolerability of this combination, FDA approved this therapy for treatment-naïve mRCC patients regardless of the IMDC risk stratification or programmed death-ligand 1 (PDL1) status. Recently, the investigators published a subgroup study for the combined intermediate/poor risk group and patients with sarcomatoid features. The observed benefits for the subgroup of improved OS, ORR, PFS, and complete response were consistent with those obtained for the total population [111].

### Avelumab plus axitinib

Axitinib also showed encouraging results with avelumab, a PD-L1 inhibitor, when compared with sunitinib monotherapy in a phase III JAVELIN Renal 101 trial. Eight hundred and eighty six randomised previously untreated patients with RCC were assigned to receive the combination therapy or the standard of care sunitinib. The primary endpoints were PFS and OS among the patients with PD-L1-positive tumours. Interestingly, the primary endpoint of longer PFS was achieved in the combination arm irrespective of the PD-L1 expression status (13.8 vs. 8.4 months). In PD-L1-positive tumour patients, the ORR was also higher than with sunitinib (55.2% vs. 25.5%). The grades 3 and 4 AEs were similar in both arms, with the most common AEs reported being hypertension, diarrhoea, fatigue, and palmar-plantar erythrodysesthesia [147]. Based on the positive results of this trial, in May 2019, the FDA approved the combination of avelumab plus axitinib to be used as a first-line treatment for patients with advanced RCC.

The treatment approaches for mRCC have been revolutionized twice, once more than a decade ago with the availability of targeted therapy and then in 2015 with the advent of immune checkpoint inhibitors. The recent new trials combined the two strategies (VEGF inhibitor and immune checkpoint inhibitors), which have proven to have significant benefits. The ongoing clinical trials with new therapeutic approaches have been reviewed in detail in the latter part of the review. In the last decade, the treatment paradigm has shifted, increasing the median survival of mRCC patients to about 33 months, and the ultimate goal of the new approaches in RCC treatment will be the long-term survival of patients.

### Lenvatinib and everolimus

A combination treatment with approved drugs is typically considered to have the potential to improve response rates and overall survival because they often exert a synergistic effect [148]. A combination of a VEGFR and mTOR inhibitor has always been an attractive therapeutic strategy in the treatment of mRCC. Many combinations were previously tried that resulted in higher toxicity without additional antitumour benefits. However, more recently, in a landmark study, a PFS advantage was observed using VEGFR and mTOR inhibitors in combination. In a phase II trial, 153 patients previously treated with VEGFR-VEGF-TKI were assigned to receive lenvatinib and everolimus either as a single agent or in combination. The primary endpoint of prolonged PFS was achieved in the combination therapy as compared to everolimus alone (14.6 vs. 5.5 months). The most common AEs were fatigue, hyporexia, and vomiting. However, the patients who received combination therapy experienced significant toxicity as compared to the single agent everolimus (71% vs. 50%). Despite the toxicity issues, the FDA approved the combination of lenvatinib plus everolimus in May 2016 for the treatment of patients with RCC who have received prior antiangiogenic therapy [149, 150]. The current first-line treatment and the subsequent treatment approaches after disease progression are outlined in Tables 2 and 3. Tables 4 and 5 describe some of the clinical trials ongoing in RCC patients.

**Table 2:** Current updated front line treatment for mRCC based on the IMDC prognostic score risk factors

Front line therapy	Limited Disease Burden/ asymptomatic	Substantial disease burden/ symptomatic
**IMDC risk**		
Good/Favourable risk	Pazopanib^a^	Nivolumab^b^ plus ipilimumab^c^
	Sunitinib^a^	Pembrolizumab^b^ plus axitinib^a^
		Avelumab^d^ plus axitinib
Intermediate and poor risk	Nivolumab^b^ plus ipilimumab^c^ Pembrolizumab^b^ plus axitinib^a^ *PD1/PD-L1 immune checkpoint inhibitor contraindications* Pazopanib^a^, Sunitinib^a^, Cabozantinib^a^	

^a^VEGFR-TKI, ^b^Anti-PD-1 antibody, ^c^Anti-CTLA-4 antibody, ^d^Anti-PD-L1

**Table 3:** Treatment approach for subsequent therapy in patients after the progression of disease with therapy failure

Disease progression with no previous exposure to anti-angiogenic therapy	VEGFR Inhibitors- Axitinib, Cabozantinib, Sunitinib, Pazopanib, Lenvatinib plus everolimus
	Nivolumab plus Ipilimumab if no previous exposure Ipilimumab
Disease progression with VEGFR inhibitor plus immunotherapy	Cabozantinib, Lenvatinib plus everolimus
Disease progression with VEGFR inhibitor without prior exposure to immune checkpoint inhibitors	Nivolumab

**Table 4:** An overview of phase III clinical trials in RCC

NCT name	Arm	Primary endpoint	Results
NCT00903175 RECORD 3	First-line Everolimus followed by second line sunitinib vs First line sunitinib followed by second line everolimus	PFS	Median PFS 21.7 months (mo.) (95%CI: 15.1–26.7) vs 22 mo. (95%CI16–29.8). Median OS: 22.4 mo. (95%CI 18.6–33.3 Vs 29.5 mo. (95% CI:22.8–33.1)
NCT00720941 COMPARZ	Control arm: Sunitinib Experimental arm: Pazopanib	PFS	HR: 1.05 95% CI: 0.90 to 1.22 OS: HR: 0.91,95% CI: 0.76–1.08
NCT00073307 TARGET	Experimental arm: Sorafenib Placebo comparator: Placebo	OS	Median PFS: 5.5mo. vs 2.8mo. HR 0.44 95% CI 0.35–0.55 *p* < 0.01. First interim analysis of OS: sorafenib reduced the risk of death (HR: 0.72; 95%CI, 0.54–0.95 *P* = 0.02)
NCT02231749 CheckMate 214	Experimental arm: Nivolumab + Ipilumab Active comparator: Sunitinib	ORR, OS, PFS	18 month OS: 75% (95%CI-70 to 78) vs 60% (95%CI: 55–65); ORR: 42% vs 27%, median PFS 11.6mo. vs 8.4 mo.
NCT01835158 Cabosun	Arm 1: Cabozantinib Arm 2: Sunitinib	PFS, OS	Median PFS 8.2 vs 5.6 mo. adjusted HR, 0.66; 95% CI, 0.46 to 0.95 *P* = 0.012 ORR 33% vs 12%
NCT02684006 Javelin Renal 101	Experimental: Avelumab plus axitinib Active comparator: Sunitinib	PFS, OS	Median PFS 13.8mo vs. 7.2 mo.HR 0.61 95% confidence interval [CI], 0.47 to 0.79; *P* < 0.001, ORR 55.2% vs. 25.5%
NCT02853331 KEYNOTE426	Experimental: Pembrolizumab + Axitinib Active comparator: Sunitinib	PFS, OS	Median PFS: 15.1 mo. vs. 11.1mo. HR: 0.69; 95% CI, 0.57 to 0.84; *P* < 0.001 ORR: 59.3% (95% CI, 54.5 to 63.9) vs. 35.7% (95% CI, 31.1 to 40.4)

*PFS* Progression-free survival, *OS* Overall survival, *HR* Hazard ratio, *ORR* Overall response rate

**Table 5:** Ongoing, active clinical trials in metastatic RCC involving small molecules in combination with other drugs

Target	NCT number	Drug	Combination drug/s or comparator drug*	Phase	Mechanism	Primary outcome	Status
HDAC Inhibitor	NCT02619253	Vorinostat	Pembrolizumab	I	Decreased proliferation, Apoptosis	MTD	Active not recruiting
	NCT03592472	Abexinostat	Pazopanib	III		PFS	
	NCT03024437	Entinostat	Bevacizumab, Atezolizumab	I/II		Safety	Recruiting
	NCT03552380	Entinostat	Nivolumab, Ipilimumab	II		Safety	Recruiting
	NCT03501381	Entinostat	IL-2 high dose*	II		PFS	Recruiting
	NCT02718066	HBI-8000 (Chidamide)	Nivolumab	I/II		Safety	Recruiting
	NCT01038778	Entinostat	Aldeslukin	I/II		Safety, dose	Active not recruiting
	NCT02890069	Panobinostat	PDR001,LCL16,everolimus	I/II		Safety	Recruiting
Glutaminase Inhibitor	NCT03163667	CB-839 (Telaglenastat)	Everolimus	II	Decreased cell proliferation and survival	PFS	Active not recruiting
	NCT03428217		Cabozantinib	II		PFS	Active not recruiting
	NCT02771626		Nivolumab	I/II		Safety, tolerability efficacy	Active not recruiting
	NCT03875313		Talazoparib	I/II		Safety, dose	Recruiting
HIF 2a Inhibitor	NCT03634540	PT2977	Cabozantinib	II	Impairs hypoxia, blocks transcription of several angiogenesis genes	ORR	Recruiting
	NCT03401788	PT2977	None	II		ORR	Active not recruiting
	NCT02974738	PT2977	None	I		Dose	Recruiting
	NCT02293980	PT2385	None	I		Dose	Active not recruiting
CD73 Inhibitor	NCT03549000	NZV930	PDR001, NIR178	I	Decreases tumour growth, promotes CTL-mediated immune response	Safety, dose	Recruiting
	NCT03454451	CPI-006	Pembrolizumab/CP-444	I		Safety, dose	Recruiting
Arginase Inhibitor	NCT02903914	CB-1158	None	I/II	Blocks Arg-1 and decreases immunosuppression	Safety	Recruiting
Her2 Inhibitor	NCT03602079	A166	None	I/II	Tumour cell apoptosis	Safety, ORR	Recruiting
CD40 Agonist	NCT03329950	CDX-1140	CDX-301,Pembrolizumab	I	Leads to activation of B cells, T cells, DC, macrophages	Safety, dose	Recruiting
	NCT03502330	APX005M	Cabiralizumab, Nivolumab	I		Safety, tolerability	Recruiting
CD137 agonist	NCT03809624	INBRX-105	None	I	T cell co-stimulation,	Safety, dose	Recruiting
	NCT02315066	PF-05082566	PF-04518600(OX-40 agonist)	I		Safety, dose	Active not recruiting
STING agonist	NCT03010176	MK-1454	Pembrolizumab	I	Production of IFNb, enhanced cross-presentation by APCs	Safety, dose	Recruiting
RIG-1 agonist	NCT03739138	MK-46212	Pembrolizumab	I	Stimulation of IFNs, enhanced anti-tumour response	Safety, dose	Active not recruiting

*MTD* Maximum tolerated dose, *PFS* Progression-free survival, *ORR* Overall response rate

## Drug resistance in RCC

The last decade has seen tremendous improvement in terms of the available treatment options for mRCC. The 5-year survival rates have improved in patients with advanced RCC over the last 10 years; yet, a large percentage of them do not respond due to innate or acquired resistance. Primary resistance is characterized as an immediate lack of response to the therapeutic compound, which occurs when the tumour cells do not express the intended target or are intrinsically resistant cells, leading to an immediate lack of response to the therapeutic compound. By contrast, acquired resistance occurs while the patient is still on treatment over the course of the disease, and tumours are able to activate the target pathways by complementary mechanisms. It is characterized as disease progression and cancer relapse after the initial tumour regression. Over the past few years, many studies have tried to examine the underlying cause of drug resistance in RCC. A comprehensive evaluation of the mechanisms of VEGF-TKI and ICI resistance will help in formulating useful knowledge about developing effective treatment strategies for patients with advanced RCC.

### Hypoxia and drug resistance

RCC is a heterogeneous tumour with widely differing blood flow conditions across tissues. Two forms of intra-tumour hypoxic conditions exist in the tumour: chronic and transient or acute hypoxia [151]. Acute hypoxia occurs due to temporary blood vessel occlusion in the inner core of tumours, whereas chronic hypoxia results from low availability of oxygen in tumour regions at a distance from blood vessels, especially in large tumours. Hypoxia can lead to impaired cellular responses, advanced and dysfunctional vascularisation along with metastasis contributes to therapy resistance by inducing cell quiescence. Clinical resistance is an important and intricate phenomenon in RCC resulting from several underlying mechanisms. Hypoxia is one of the key factors in RCC, correlating with poor prognosis in RCC progression and affecting activities at the cellular level, resulting in resistance of the tumour cells to VEGF-TKIs and ICIs.

ICI-mediated antiangiogenic therapy suppresses the production of proangiogenic factors or inhibits their binding to their respective receptors, subsequently halting angiogenesis; hence, they were approved for the treatment of mRCC [152]. However, sustained treatment with antiangiogenic therapy consequently leads to the development of secondary hypoxia caused by decreased vasculature due to drug treatment. Tumour cells can adapt to sustained hypoxia through coordinated and complex intracellular signalling responses, resulting in several VEGF- and PDGF-independent proangiogenic factors, such as EGFR, PIGF, FGF2, erythropoietin (EPO), TGF-α, ΙL-6, IL-8, which induce acquired resistance and therapy failure, most importantly, activation of the HIF pathway [153].

Studies have shown overexpression of FGF 1/2, ephrin A1 and A2 (EFNA1/2) and angiopoietin 1 and 2 (Ang 1/2) as a direct result of hypoxia induced by antiangiogenic treatments. FGF can prompt endothelial cells to proliferate and form endothelial tubules in the presence of VEGF-TKI [154]. FGF/FGFR pathway regulates intracellular signalling cascades, such as MAPK/ERK, PI3K/Akt, STAT, inositol triphosphate, and diacylglycerol protein kinase C, and aberrant signalling in these pathways is linked to the development of sunitinib resistance [155]. Inhibition of these targets with lenvatinib plus everolimus has demonstrated superior activity in patients previously treated with sunitinib [149]. The Ang/Tie signalling pathway is another important and alternative angiogenic pathway in RCC. The pathway modulates endothelial cell survival and vascular maturation. Ang 2 levels are decreased in patients responding to sunitinib therapy; however, it is increased when the patients start showing resistance to sunitinib [156]. CovX-bodies (protein-antibody construct) are a new class of biotherapeutics that have demonstrated decreased tumour vessel density when combined with sunitinib and sorafenib [157].

Recently, the interaction between the immune system and angiogenesis has gained momentum. IL-6, known to cause resistance to IFN-α, has also been shown to be an inducer of VEGF-TKI resistance and a potent activator of AKT-mTOR and signal transducer and activator of transcription 3 (STAT3) pathway, along with HIF-2α, all of which leads to VEGF expression [158]. IL-8, an important proangiogenic factor, is known to have high expression in patients resistant to VEGF-TKIs, which enhances angiogenesis via autocrine activation of VEGFR-2 and proliferation of endothelial cells [159]. Both IL-6 and IL-8 are poor prognosis indicators in RCC and can serve as a therapeutic target to reverse VEGF-TKI resistance, as shown in several studies [158–160]. Similarly, PIGF, a VEGF homolog known to increase angiogenesis by binding to VEGFR-1 is expressed by tumour, proangiogenic, inflammatory, stromal, and endothelial cells. It also enhances the expression of VEGF-A, FGF2, PDGFβ, and MMPs [154, 161].

Hypoxia also modulates ICI-mediated intrinsic resistance to therapy by selecting aggressive CSC-like RCC subpopulations of cells that are more malignant and unresponsive to antiangiogenic treatment [44]. Tumour-associated hypoxia also increases the expression of P-glycoprotein membrane exporter (P-GP), which is responsible for cellular sequestration of many VEGF-TKIs, including sunitinib, pazopanib, and sorafenib [162].

Resistance to ICIs also occurs because hypoxia can drive immunosuppression directly and indirectly, which can affect almost all steps of classic antitumour immune cell responses. The acidic environment in the tumour, due to the rapid consumption of oxygen together with hypoxic conditions, excessively inhibits the activation, proliferation, and cytotoxicity of T cells. PD-1 and its interaction with PD-L1 results in suppression of the T cells to suppress their cytotoxic activities. In ccRCC, hypoxia via HIF-2α stabilizes and upregulates PD-L1 expression [152]. HIF-1α, by contrast, leads to the overexpression of PD-L1 in immune cells such as myeloid-derived suppressor cells (MDSCs) and macrophages, which in turn negatively regulate cytotoxic T cells [163]. Along with PD-L1, hypoxia also affects other immune checkpoint inhibitors, such as the V-domain Ig suppressor of T cell activation (VISTA). VISTA is overexpressed in a hypoxia-dependent manner in MDSCs and dendritic cells (DCs), leading to suppression of T cell multiplication and cytotoxic killing of tumour cells. Hypoxia can also indirectly lead to the production of tumour-associated macrophages (TAMs), cytotoxic T lymphocytes (CTLs), and Tregs. Hypoxic MDSCs are more immunosuppressive due to the overproduction of arginase and nitric oxide (iNOS), all of which lead to T cell inactivation and tumour progression [164]. CTLA-4 is also affected by hypoxia by promoting binding with its natural receptors CD80 and CD86, thereby downregulating immune responses [165].

### Resistance to antiangiogenic agents

Hypervascular tumours such as RCC are dependent on increased production of growth factors including VEGF and PDGFβ. In 2008, Gordon et al. suggested that deregulated stimulation of HIF-1α and HIF-2α genes results in the activation of different oncogenes [166]. It was noted that the subgroup with only HIF-2α expression demonstrated primary resistance to the antiangiogenic drugs. Other mechanisms that are known to be associated with primary resistance include increased production of B cell lymphoma-2 (Bcl-2) and/or B cell lymphoma-extra-large (Bcl-XL) proteins leading to inhibition of apoptosis and overexpression of enhancer of zeste homolog 2 (EZH2) and epigenetic modification of its promoter region [167, 168].

Emerging studies suggest the role of five different important processes involved in evasive resistance to antiangiogenic therapies: a) lysosomal sequestration of the drugs leading to its low bioavailability, b) tumour invasiveness and EMT, c) increased pericyte coverage of tumour vessels, d) bone marrow-derived proangiogenic inflammatory cell recruitment, and e) resistance through single nucleotide polymorphisms (SNPs) and microRNAs (miRNA).

#### Lysosomal sequestration of drugs

Sunitinib is a weak base, and its hydrophobic structure allows easy passage through the lysosomal plasma membrane. Once inside the acidic environment of the lysosomal structures, sunitinib becomes charged and is trapped and retained inside the lysosome. This process is known as sequestration and protects the cells from its antiangiogenic effects despite the high intracellular concentrations of the drug. Interestingly, lysosomal sequestration is a reversible process [169, 170]. Studies on sunitinib-resistant RCC tumour cell lines have shown that sensitivity to the drug was regained and the lysosome capacity reversed after the cells were cultured without sunitinib [171].

#### Tumour invasiveness and EMT

Increased tumour invasiveness and initiation of malignant phenotype helps tumours to adapt to antiangiogenic therapy. In a preclinical mouse model of glioblastoma, tumour invasiveness was observed despite the downregulation of VEGF, HIF-α, and matrix metalloproteinases (MMP-9). Induction of EMT genes activates signal cascades responsible for drug resistance, metastasis, and angiogenesis [172]. Reversal to an epithelial phenotype along with sensitivity to the antiangiogenic drugs has been demonstrated in RCC and can be considered a potential therapeutic strategy [53].

#### Increased pericyte coverage of tumour vessels

Pericytes are cells that are wrapped around blood vessels and express VEGF and other factors to support the proliferation and migration of endothelial cells. An increased number of pericyte-generated microvessels have been directly linked with aggressive ccRCC and resistance to therapy [173]. Pericytes are found to be critical for maintaining the tumour vasculature and increased angiogenesis in the absence of VEGF signals and therefore are perceived as potential new therapeutic targets [174, 175].

#### Bone marrow-derived proangiogenic inflammatory cell recruitment

It is widely accepted that antiangiogenic therapy causes hypoxia, which in turn stimulates proangiogenic factor production in tumours as well as recruits different bone marrow-derived cells (BMDCs). These BMDCs include CD11b-positive myeloid-derived suppressor cells (MDSC), proangiogenic tumour associated macrophages (TAM), VEGFR1-positive hemangiocytes, and circulating endothelial cells [176–179]. The proangiogenic and immunosuppressive nature of these BMDCs may be responsible for drug resistance in patients.

#### Resistance through single nucleotide polymorphisms (SNPs) and microRNAs

SNPs are DNA sequence variations of a single base pair that achieve a population frequency of a minimum of 1%. There are SNPs located on genes that regulate the pharmacokinetics and pharmacodynamics of the VEGF-TKIs and hence may contribute to the development of VEGF-TKI resistance [180]. SNPs in these genes can be used as predictive markers of drug resistance or efficacy [181]. CYP3A4/5 are the key enzymes in the metabolism of sunitinib. SNPs in CYP3A5 positively regulate the metabolism of sunitinib, leading to an increased metabolism of sunitinib and are often associated with increased PFS in patients [182]. By contrast, SNPs in ligand-activated nuclear receptors NR112 and NR113 negatively regulate CYP3A4 expression and are associated with decreased PFS [183]. SNPs in pharmacodynamic factors of the drug, such as VEGF-R1–3, can also lead to sunitinib resistance.

miRNAs are noncoding RNAs that play a critical role in cancer progression by silencing tumour suppressor genes. In RCC, several miRNAs have been identified. Recently, overexpression of miRNA-15b was linked to sunitinib resistance [184]. In a mi-RNA profiling study in resistant RCC cell lines, miRNA-575, miRNA-642b-3p, and miRNA-4430 were overexpressed [185].

### Activating bypass pathways

**Fig. 5:**
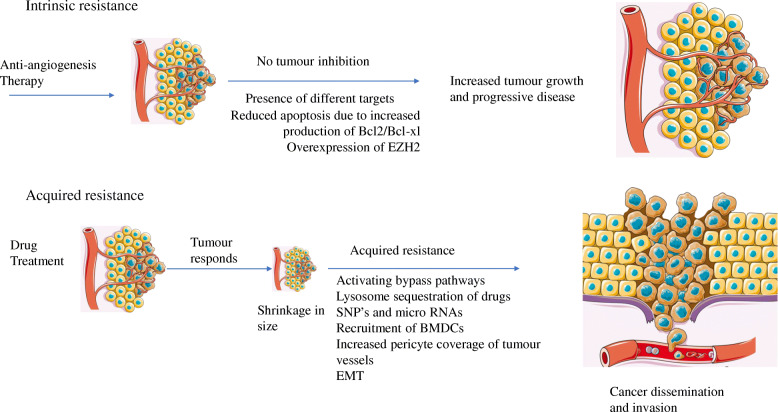
Tumour resistance mechanisms against anti-angiogenic treatments. The upper image shows the various innate resistance mechanisms, which include a) presence of different targets, b) reduced apoptosis due to overproduction of Bcl2/Bcl-xl, and c) over expression of EZH2. In the lower image, the tumour responds initially in response to therapy by shrinking in size but undergoes acquired resistance which facilitates cancer re-growth. Some of the resistance mechanisms are a) initiation of alternative or complementary pathways, b) lysosomal sequestration of the drugs, c) SNPs and micro RNAs, d) recruitment of BMDCs, e) increased pericyte coverage of tumour vessels, f) EMT and g) establishment of new-pro-angiogenic pathways

Pathway bypass mechanisms confer drug resistance by recruiting alternate effector pathways to sustain oncogenic transcription and translational output. Phosphatase and tensin homolog (PTEN) is a tumour suppressor gene that negatively regulates the PI3K/Akt/mTOR pathway [186]. Mutations in PTEN lead to a continuous expression of AKT/mTOR signalling pathway. Although PTEN mutations are rare in RCC, a non-negligible number of studies have shown that PTEN is mutated and downregulated in many RCC patients [186]. Figure 5 summarizes the resistance pathways to antiangiogenic therapies.

## Resistance to immune checkpoint inhibitors

For the immune system to destroy tumour cells and for the immune checkpoint inhibitors to work efficiently, three important steps of the cancer-immunity cycle have to occur: 1) antigen presentation and T cell activation, 2) T cell trafficking and tumour infiltration, and 3) T cell killing activity within the TME. Immune evasion can occur at any of the above steps and can lead to primary or adaptive resistance in patients receiving immunotherapy.

### Lack of T cell priming and impaired antigen presentation

The inability of the immune system to elicit a response against tumour antigens can result from the lack of tumour antigens that can be recognised as foreign or the lack of sufficient antigen presentation by the antigen-presenting cells [187]. The primary reason for low immunogenicity in patients may result from a low mutational burden causing in inadequate tumour-associated antigens presentation by DC that fails to initiate a CD8^+^ response in the patients. RCC has a relatively low mutational burden [188], and this can render RCC tumours non-responsive to ICI treatments. Lack of antigen presentation can also be attributed to mutations that affect the structure of major histocompatibility complex (MHC), proteasome, and transporters associated with antigen processing.

Loss of beta-2-microglobulin can lead to impaired MHC class I expression. This loss causes a subsequent decrease in antigen presentation via MHC class I, leading to reduced detection of the tumour cells by CD8^+^ cells and rendering tumours less sensitive to T cell infiltration. Antigen presentation and recognition with the help of MHC can also be affected by epigenetic changes such as histone acetylation and hypermethylation.

Functional antigen presentation by mature DCs is an absolute requirement for T cell activation. Mature DCs have improved capacity to stimulate T cells through high expressions of cytokines and expression of MHC class I/II and a variety of costimulatory molecules, such as CD80, CD86, and CD40, which are key to the processes of T cell priming [189]. Immune checkpoint inhibitors rely heavily on the cross-presentation of tumour antigens, and the process is hampered when the DCs present in the TME do not function effectively. Hypoxia increases lactate levels, and a decrease in pH can suppress DC maturation and function [190]. STAT3 is also known to promote crosstalk between the tumour and immune cells and induces S100 calcium binding protein A9, which suppresses the maturation of DCs by blocking their responsiveness to local danger signals. STAT3 is also responsible for the induction of immunosuppressive factors such as IL10, regulatory T cells (Treg), and transforming growth factor beta (TGF-β), all of which may impair the maturation and normal function of DCs [190, 191].

### Decreased T cell activity in the tumour microenvironment (TME)

A recent RCC study showed that TME has an impact on the way cancer progresses and resists ICI therapies [192]. Loss of function mutations of Janus kinase1/2 (JAK1/JAK2), which is responsible for the antitumor activity of IFN-γ, can lead to tumours being refractory to immune checkpoint inhibitors. The interaction of tumour cells with immune cells can have multiple outcomes. Certain interactions can impair the antitumour activity of the ICIs despite adequate antigen presentation and T cell infiltration. Either Tregs inhibit T cell activity directly via cell-to-cell contact or indirectly by secreting inhibitory molecules such as IL-10, IL-35, and TGF-β, all of which can suppress CD8 + T cell infiltration into tumours and its associated functions [191, 193]. MDSCs can suppress T cell activity by a series of various actions, including production of ROS, arginase, IL-10, nitrosylation of chemokines, and depleting nutrients such as cysteine and tryptophan, which are vital nutrients for T cells [194]. Studies have shown that depleting or impairing Tregs or MDSCs restores the anticancer activity of ICIs [191, 195, 196].

Upregulation of immune checkpoint markers has been linked to decreased T cell killing in TME. Increased expression of PD-L1 can lead to decreased functioning of cytotoxic T cells and apoptosis, leading to tumour progression [197]. Indoleamine 2,3-dioxygenase (IDO1) and lymphocyte activation gene 3 (LAG3) can lead to negative regulation of T cells by activation of MDSCs in a Treg-dependent process and by leading to a state of immune exhaustion in the TME, respectively [198, 199]. Tumour cells expressing PD-L1 and T cell immunoglobulin mucin (TIM-3) can downregulate T cell functions [200].

### Absence of T cells in the tumour microenvironment

**Fig. 6:**
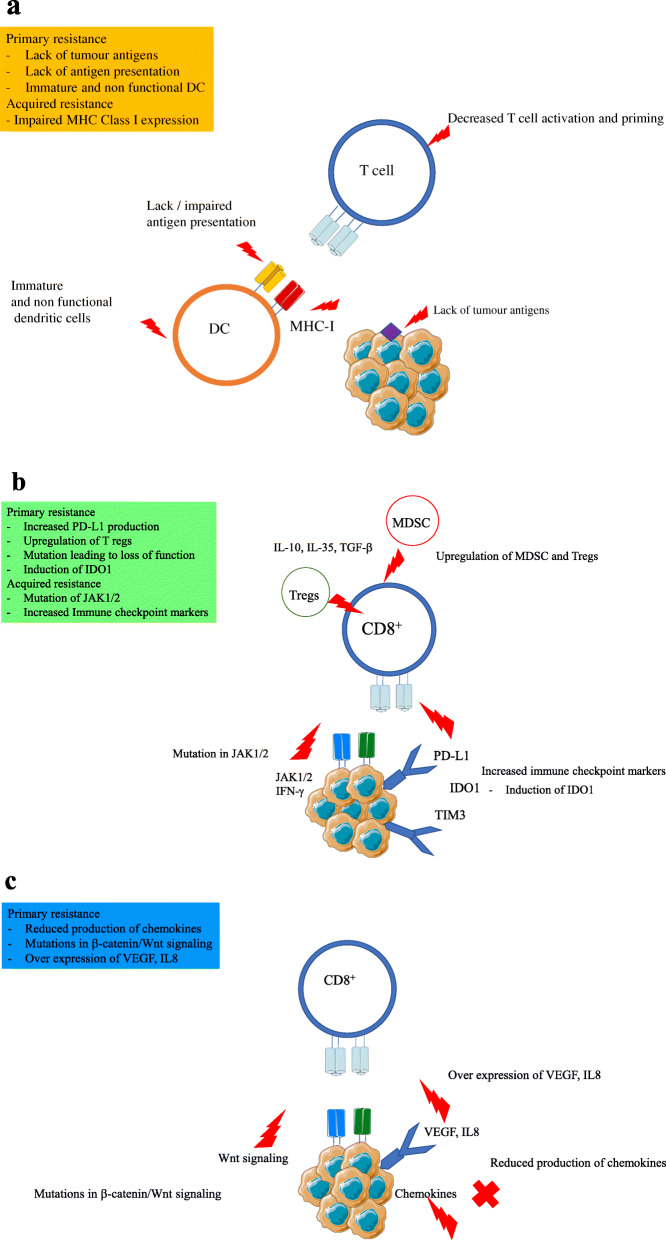
Resistance to ICI therapy. Various putative or acquired immune escape mechanisms can result in tumour evading the immune checkpoint inhibitor therapy. These include; a) Lack of T cell priming through impaired antigen presentation via inadequate DC maturation or through appropriate MHC1 expression; b) decreased T cell activity in the tumour microenvironment through increased infiltration of Tregs, expression of T cell suppressive molecules on tumour cells such as IDO, PD-L1, or mutational changes in JAK2 pathway, loss of tumour suppressor genes such as PTEN or increased expression of T cell exhaustion markers such as TIM-3, PD-1, etc.; c) lack of T cell infiltration in the tumour microenvironment due to enhanced LDH accumulation, low expression of T cell migratory chemokines, increased expression of tumour suppressive cytokines such as TGF-β, IL-6, IL-8, etc.

Mutations within the tumour can result in the inhibition of T cell recruitment to the tumour microenvironment. Mutations that lead to an increase of β-catenin/Wnt signalling in tumours ultimately leads to reduced CD8^+^ T cells and CD103^+^ infiltration in the tumour [201]. Expression of C-X-C motif chemokine receptor (CXCR3) is important for T cell trafficking and function [202]. Epigenetic alterations, including histone modification and DNA methylation in tumour cells, repress chemokines, thereby allowing tumour cells to evade T cell trafficking [203]. Other mutations in the MAPK pathway, leading to overexpression of VEGF and IL-8 have also been detected. These cytokines inhibit T cell recruitment in the tumour. Mutation of the tumour suppressor PTEN gene is also linked to decreased CD8^+^ T cell infiltration owing to the inhibitory activity of overexpressed VEGF. Figure 6 summarizes some mechanisms of ICI resistance in RCC patients.

### Composition of gut microbiome

Current studies have revealed the potential role of the gut microbiome in modulating primary ICI resistance in RCC [204, 205]. The composition of the gut microbiome may affect the antitumour activity of the ICIs [152]. Greater microbial diversity correlates with improved response to ICIs [152]. Antibiotics can greatly alter gut biodiversity. A study tested the effect of antibiotics on patients with RCC who had previously received PD-1/PD-L1mAb. A shorter PFS and OS was observed in the antibiotic-treated patient group. Further, the presence of *Akkermansia muciniphila* bacterial species in the gut was associated with excellent clinical outcomes in RCC patients. The tumour suppressor activity may be partly due to the production of the short-chain fatty acids, propionate and acetate, by the commensal bacteria. Propionate is the ligand of G-protein-coupled receptor 41, which mediates tumour cell apoptosis [204]. Another study showed that antibiotic therapy was associated with reduced clinical outcomes with ICIs in RCC and suggested modulation of antibiotic therapy-related dysbiosis and gut microbiota as a strategy to improve the clinical benefits of ICI treatment [205]. In summary, multiple resistance mechanisms could be at play in mRCC patients treated with VEGF inhibitors and ICIs as monotherapy.

## Vaccines and viruses

Several vaccines that target tumour-specific antigens called neo-antigens are now under development for RCC. The trials undertaking vaccines are either as monotherapy or in conjunction with immune checkpoint inhibitors. Currently, three types of therapeutic cancer vaccines are being researched in different cancers: autologous and allogenic vaccines, DC vaccines and protein/peptide-based, genetic vaccines (DNA/RNA) [206]. A phase I study of recombinant adenovirus encoding GMCSF-CAIX fusion gene transduced autologous dendritic cells in RCC showed encouraging results with regards to its safety in patients. Oncolytic viruses are promising alternatives that specifically target cancer cells, infecting and replicating in them. The viruses are modified to use cancer cell machinery to induce transgene expression, resulting in the apoptosis of tumour cells. All the clinical trials using vaccines and viruses in RCC are described in detail in Table 6.

**Table 6:** Summary of active clinical trials applying vaccinal and oncolytic virus strategies

NCT number	Agent/Drug	Vaccine type	Combination drug/s	Phase	Mechanism	Primary outcome	Status
NCT02950766	NeoVax	Peptide-based	Ipilimumab	I	Personalised neoantigen vaccine	Safety, dose	Recruiting
NCT03715985	EVAX-01-CAF09b	Peptide-based	anti-PD-1, anti-PD-L1	I		Safety, efficacy	Recruiting
NCT00458536	DC tumour fusion	Dendritic cell	GM-CSF	I/II	Improving antigen presentation	Safety	Active not recruiting
NCT03548467	VB10.NEO	DNA Plasmid	Bempegaldesleukin	I/II	Improved immune response elicited by CD4^+^ and CD8^+^ T cells	Safety	Recruiting
NCT03633110	GEN-009	Autologous	Nivolumab/Pembrolizumab	I/II	Elicit CD8^+^ T cell response	Safety	Active not recruiting
NCT03294083	Pexa-Vec	Oncolytic vaccinia virus	REGN2819	I	Stimulate immune response, replicates in and lyses tumour cells	ORR, safety, dose	Recruiting

## Novel therapeutic strategies currently being tested for mRCC

Research in RCC is continuously evolving, and more understanding of the molecular characteristics and resistance mechanisms in RCC tumours is being discovered. Clinical trials involving novel strategies are being carried out with the primary objective of improving the clinical outcome and expanding treatment options for patients with mRCC.

### Novel therapies targeting tyrosine kinases under investigation

Preliminary studies on savolitinib, also called volitinib, a highly selective MET inhibitor, showed anti-tumour activity in pRCC [207]. pRCC is a MET-driven tumour and currently has no specifically approved treatment. SAVOIR, a randomised phase III trial, is evaluating the efficacy and safety of savolitinib compared with sunitinib (NCT03091192). The role of Activin receptor-like kinase (ALK) is well known in modulating angiogenesis. Dalantercept, a known ALK inhibitor, showed promising results in a phase I trial; however, it did not appear to improve the treatment-related outcomes in RCC patients despite being well tolerated [208]. Crizotinib, an ALK and MET inhibitor, was well tolerated in advanced metastatic pRCC and achieved long-lasting disease control in a cross-tumoural phase II trial, CREATE. The preliminary results were published in 2017, and the study is expected to be completed in 2020 (NCT01524926). In a phase II trial study, savolitinib is being compared to cabozantinib S-malate, crizotinib, and sunitinib malate in metastatic pRCC (NCT02761057). A VEGFR and PDGFR inhibitor, vorolanib in combination with everolimus, is being evaluated in a phase II/III trial, CONCEPT study (NCT03095040). Furthermore, ibrutinib, a Bruton tyrosine kinase (BTK) inhibitor, showed a decrease of the renal mass in a case study involving a 66-year-old male referred for a left renal mass along with newly diagnosed CLL. Currently, there are two-phase I/II clinical trials investigating the use of ibrutinib in mRCC (NCT02899078 and NCT02599324). Ibrutinib shows its effect through inhibition of IL2-inducible T cell kinase, leading to a shift in the ratio between T helper 1 and T helper 2 T cells, thereby enhancing antitumour activity. They are also known to inhibit PD-1 or CTLA-4 [209].

### Novel immunomodulatory approaches

Modulation of these immune responses remains a very fascinating therapeutic approach in mRCC. Most of the focus has remained on PD-1/PD-L1 and CTLA-4. However, few other immunomodulatory pathways and molecules are being exploited to improve the overall response of mRCC patients. Some recent strategies for ICI treatment in RCC are described below.

LAG-3 exerts immune homeostasis by suppressing T cell functions and is normally expressed by exhausted tumour-infiltrating lymphocytes. It interacts in synergy with PD-1 to inhibit immune responses. Currently, few trials are investigating the action of LAG-3. LAG525, an anti-LAG-3 antibody, is being used as a single therapy and in combination with PDR001 and anti-PD1 (NCT02460224). A phase II trial FRACTION-RCC is investigating relatlimab, another anti-LAG-3 antibody in combination with nivolumab (NCT02996110). A phase I study, DUET-4, which evaluates XmAb®22,841, a bispecific antibody targeting CTLA-4 and LAG-3, is being undertaken and is expected to be completed in 2027 (NCT03849469).

IDO-1 is an immunomodulatory enzyme that converts tryptophan to kynurenine. It suppresses T and NK cells and promotes the proliferation of Tregs and MDSCs. The overexpression of IDO-1 in tumours leads to the depletion of tryptophan and increased production of kynurenine in the TME. These changes result in ineffective T effector cell activity and an increased immunosuppressive TME, allowing the tumour cells to escape immune surveillance. Epacadostat and Linrodostat (BMS-986205) are the two IDO-1 inhibitors currently under investigation in RCC under separate clinical trials. NCT03260894, NCT02178722, and NCT03277352 are all in combination with pembrolizumab and other drugs. NCT02996110 is in combination with nivolumab.

Hypoxia and cellular damage in cancer lead to the accumulation of adenosine in TME. Extracellular adenosine, along with its G-protein-coupled adenosine receptors, mediates immunosuppressive activity through various mechanisms, such as stabilizing Tregs, rendering CD8 T cells ineffective, downregulating IL2, and inhibiting TCR signalling. Some clinical trials are underway investigating adenosine receptor inhibitors alone or in combination with other drugs: AB928 (NCT03629756) and CPI-444 (NCT03454451, NCT02655822).

V-domain Ig suppressor of T cell activation (VISTA) impedes T cell activity, and blocking it has been shown to improve T cell activity and inhibit T cell immunosuppressive functions. CA-170, a small molecule VISTA inhibitor that also targets PD-L1/2, is being investigated in a phase 1 trial in RCC and other advanced solid tumours. This trial aims to assess the dose-limiting toxicity in the first treatment cycle (NCT02182875).

Co-stimulatory immune checkpoint inhibitors are also in ongoing trials in mRCC. Agonistic antibody of glucocorticoid-induced TNF receptor (GITR) and OX40 (CD137) and co-stimulatory receptors of TNF superfamily are under evaluation in various clinical trials. Expression of GITR enhances antitumour activity by decreased immunosuppression and enhanced co-stimulatory function of CD8^+^T cells, whereas OX40 enhances the proliferation and survival of CD4^+^ and CD8^+^ T cells. INCAGN0186 is an agonistic anti-GITR antibody, while PF-04518600 is an agonistic antibody of OX40. The compounds are being investigated alone (NCT02697591 and NCT02315066, respectively) or in combination with other already approved drugs, such as nivolumab + ipilimumab (NCT03126110) and axitinib (NCT03092856).

Chimeric-antigen receptor (CAR) T cells are genetically engineered T cells that have recently entered the therapeutic testing horizons of RCC. The T cells are tailor-made to have antigen-binding receptors along with signalling domains needed for T cell activity. CAR-T cells have shown promising results in haematological malignancies, but the same has yet to be obtained in solid tumours like RCC, especially because of the immunosuppressive TME prevalent in these tumours. However, in a recent pre-clinical study, CAIX-specific CAR-T cells in combination with sunitinib showed synergistic effects against mRCC models [210]. CAR-T cells are currently being tested targeting a plethora of antigens, namely CD70 (NCT02830724), c-MET (NCT03638206), VEGFR2 (NCT01218867), ROR2, and AXL (NCT03393936). There are now second-generation CAR-T cells that have additional co-stimulatory receptors (CD28 and/or 4-1BB).

### Combination therapy (target therapy and immunotherapy)

Pathways affecting angiogenesis and immune responses are intertwined and can positively or negatively affect each other. Of no surprise is the proven success of combining ICIs and target therapy drugs as an emerging approach in several clinical trials. The current ongoing phase III trials combining immune checkpoint inhibitors and target therapy are detailed in Table 7.

**Table 7:** Active Phase III clinical trials with novel therapeutic drugs in mRCC

NCT name	Drug/procedure combinations	Primary outcome
NCT03055013 PROSPER	Nephrectomy vs. Perioperative Nivolumab	RFS
NCT03592472 RENAVIV	Pazopanib + Placebo vs. Pazopanib + **Abexinostat**	PFS
NCT03260894	Sunitinib/Pazopanib vs. Pembrolizumab + **Epacadostat**	ORR
NCT03288532 RAMPART	**Durvalumab** vs Durvalumab + Tremelimumab	DFS
NCT03142334	Pembrolizumab (Adjuvant setting)	DFS
NCT03138512 CheckMate 914	Nivolumab + Ipilimumab (Adjuvant setting)	DFS
NCT03091192	**Savolotinib** vs. Sunitinib	PFS
NCT03024996 IMmotion 010	Atezolizumab (Adjuvant setting)	DFS

### Other small molecule drugs

Currently, immune checkpoint inhibitors are also being tested in combination with many other small molecules. In particular, a few different histone deacetylase (HDAC) inhibitors are being tested. HDAC1 and HDAC2 are important for the growth and survival of RCC cells. Inhibition of HDACs can lead to apoptosis and reduction of proliferation of the RCC cells [211]. HDAC inhibitors entinostat and panobinostat are currently being investigated in combination with immune checkpoint inhibitors nivolumab plus ipilimumab and PD-1 inhibitor PDR001 respectively. A phase III clinical trial RENAVIV is currently investigating Abexinostat, a deacetylase inhibitor, along with pazopanib as a first-line drug for mRCC and is expected to be finished by 2022 (NCT03592472).

## Future direction and conclusion

The number of published reviews on RCC in the literature are enormous. However, most of them focus on specific aspects of RCC, such as the biology, directed towards specific molecular mechanisms for example hypoxia, EMT/CSC, or emphasize on the failure of past treatments and the pros and cons of present treatment methods. There is, however, a lack in the literature of studies that consolidate findings on basic biological changes leading to RCC development, which can be interrogated clinically to identify drug targets forming the basis of clinical trials and subsequent treatments. In this review, we have tried to address these issues, attempted to provide a holistic view on the fundamental biological changes regulating RCC development, progression and combined that information with the basis of therapy resistance, development of clinical trials and subsequent treatments. Our other rationale for this review was to consolidate the information in the literature about the molecular and biological changes leading to RCC progression and justify that with the data generated in our laboratory on patient samples. In addition, effort was made to update the readers on the mechanistics and pros and cons of current treatment protocols, information on current clinical trials, and the conceptualisation of the evolving RCC field in relation to precision medicine.

With the recent understanding of RCC pathogenesis, many new therapeutic strategies are being developed, and a few of them including immunotherapies targeting programmed cell death (PD-1)/programmed death-ligand 1 (PD-L1), as single agents or in combination with anti-CTLA-4 monoclonal antibodies or a multi-target VEGF-TKIs have gained FDA approval as treatment regimens in clinical settings. Such progress has improved the overall survival of mRCC patients. However, the overall response rates achieved through immune therapy and combination treatments still remains as low as ~ 20–30% and is associated with substantial toxicity profiles, which needs improvement to achieve better treatment results [212–214]. One approach to achieve higher response rates in patients would be to target multiple checkpoint inhibitors besides the commonly known ones in combination with TKIs. However, this may result in greater toxicity [142, 215]. Hence, there is an urgent need to identify alternative combination strategies, which would improve clinical outcomes in patients without the significant risk of toxicities.

As discussed above, HIF-associated hypoxia is the hallmark of RCC initiation and progression. Hypoxia not only induces genomic instability but also accelerates EMT/metastases, defects in apoptosis/autophagy, induction of CSCs and resistance to therapies. Hypoxia generates an inflamed pro-angiogenic, immunosuppressive TME, which boosts the malignant cells to undergo metastatic progression and therapy resistance. Hence, specific targeting of hypoxia using hypoxia-targeted drugs may achieve better results in RCC treatment. In that context, trials using hypoxia targeting signalling molecules are currently being exploited in cancer therapy [216, 217].

Further to that, from the hypoxia standpoint, caution should be taken in using maximum tolerated dose (MTD) of traditional anti-angiogenic drugs as it may successfully shrink the tumours but induce secondary hypoxia, accelerating hypoxia-associated tumourigenesis and compromising anti-tumour immunity by priming immunosuppressive cells infiltration in tumours [218, 219]. In that context, an elevated concentration and extended period of anti-VEGF therapy treatment was associated with decreased oxygen supply associated with hypoxia in tumours [220, 221]. Nevertheless, a proper use and timing of the anti-angiogenic drugs can revert the hypoxia process by normalizing the tumour vasculature by intervening with oxygen delivery [222, 223]. Hence, dosage of anti-angiogenic drugs and the frequency of its administration is key for successful anti-angiogenic treatment. A lower concentration of anti-angiogenic drugs, as low as one-quarter of the conventional dose, has shown sustained vascular normalization in preclinical models [220, 224]. Clinical studies in patients with glioblastoma and breast cancer have confirmed these results, by showing that a decreased dose of anti-VEGF (< 3.6 mg/kg/week) after cytoreductive surgery provides improved survival compared to high dose of anti-VEGF at 5 mg/kg/week [225, 226]. In addition, combination of anti-angiogenesis and anti-immune therapy studied in pre-clinical models as well as in clinical settings have shown immense potential as future therapeutics [227–229]. Hence, the treatment landscape of mRCC holds promise by optimising low doses of combination of anti-angiogenic and immune-directed therapies essential to tackle the complex hypoxia-oriented TME.

Despite the above developments, a wide research gap still exists in understanding individual patient tumour biology and alignment of that with the choice of appropriate drug for treatment. Unlike most solid tumours, there are currently no biomarker driven drug approvals in RCC [230]. Immune and other targeted therapies are prescribed to RCC patients depending on specific risk scores [231]. In addition, no reliable biomarker exists to indicate which group of patients will benefit from specific immune therapies [232]. Even though cytoreductive nephrectomy is common in RCC patients, ~ 30% of metastasis-free patients develop recurrent cancer after cytoreductive surgery within a short time frame. This suggests that there is a need for the use of adjuvant therapy, which will keep the microscopic tumour under check after cytoreductive surgery. In the adjuvant settings, TKIs on their own have not proven satisfactory, suggesting a change of treatment paradigm, which relies on immunotherapy with or without TKIs in moderate-high risk patients. However, in that scenario establishment of valid predictors for immunotherapy treatment response is essential [232]. As a single biomarker for patient selection has not proven successful, a panel of biomarkers based on components such as PD-LI expression in tumours, degree of tumour infiltrated T cells, expression of exhaustion markers on lymphocytes, tumour mutational burden, etc. may provide better prediction of patient’s response to immune and related therapies. Furthermore, a more improved understanding of the biology of different subtypes of RCC based on the data collected by large public datasets such as TCGA, TRACERx Renal, etc. may provide identification of biomarker-oriented approaches necessary for the stratification of patients for specific treatment modalities [230]. This may guide clinicians to tailor best-personalized treatment to patients.

## Data Availability

The datasets presented in the review are not publicly available as it is part of a PhD thesis, which is in progress. However, if required the data can be obtained from the corresponding author on a reasonable request.

## Abbreviations

VEGF-TKI: Tyrosine kinase inhibitors
RCC: Renal cell carcinoma
ICI: Immune checkpoint inhibitors
ccRCC: Clear cell renal cell carcinoma
pRCC: Papillary RCC
chRCC: Chromophobe RCC
*VHL*: von-Hippel-Lindau gene
pVHL: von-Hippel Lindau protein
HIF: Hypoxia inducible factor proteins
LDH: Lactate dehydrogenase
PDK: Pyruvate dehydrogenase kinase
GLUT1: Glucose transporter 1
HK: Hexokinase
EGFR: Epidermal growth factor receptor
p53: Tumour protein 53
mTOR: Mammalian target of rapamycin
EMT: Epithelial-mesenchymal transition
OS: Overall survival
PFS: Progression-free survival
IL6: Interleukin 6
IL8: Interleukin 8
TNF-α: Tumour necrosis factor α
miRNA: Micro-RNA
CSC: Cancer stem cells
TGFβ: Transforming growth factor β
CD: Cluster of differentiation
mRCC: Metastatic RCC
FDA: Food and Drug Administration
IFN a: Interferon a
VEGF: Vascular endothelial growth factor
VEGFR: Vascular endothelial growth factor receptor
mPFS: Median PFS
ORR: Overall response rate
IMDC: International Metastatic RCC Database
AE: Adverse effects
PD-1: Programmed cell death protein − 1
PD-L1: Programmed cell death ligand − 1
TME: Tissue microenvironment
MMP: Matrix metalloproteinases
BMDC: Bone marrow-derived cells
TAM: Tumour associated macrophages
SNP: Single nucleotide polymorphisms
PTEN: Phosphatase and tensin homolog
STAT3: Signal transducer and activator of transcription 3
MDSC: Myeloid-derived suppressor cells
IDO1: Indoleamine 2,3-dioxygenase
LAG3: Lymphocyte activation gene 3
TIM3: T-cell immunoglobulin mucin
